# Accuracy of Point-of-care Ultrasound in Diagnosing Acute Appendicitis During Pregnancy

**DOI:** 10.5811/westjem.2022.8.56638

**Published:** 2022-10-23

**Authors:** Désirée Abgottspon, Katharina Putora, Janis Kinkel, Kinga Süveg, Bernhard Widmann, René Hornung, Bruno Minotti

**Affiliations:** * Cantonal Hospital of St. Gallen, Department of Obstetrics and Gynecology, St. Gallen, Switzerland; † Cantonal Hospital of St. Gallen, Division of Radiology and Nuclear Medicine, St. Gallen, Switzerland; ‡ Cantonal Hospital of St. Gallen, Department of General, Visceral, Endocrine and Transplantation Surgery, St. Gallen, Switzerland; § Cantonal Hospital of St. Gallen, Department of Emergency Medicine, St. Gallen, Switzerland; ¶ University Hospital Basel, Department of Emergency Medicine, Basel, Switzerland

## Abstract

**Introduction:**

Acute appendicitis is the most common non-obstetrical surgical emergency in pregnancy. Ultrasound is the imaging tool of choice, but its use is complicated due to anatomical changes during pregnancy and depends on the clinician’s expertise. In this study, our aim was to investigate the diagnostic accuracy of point-of-care ultrasound (POCUS) in suspected appendicitis in pregnant women.

**Methods:**

We conducted a retrospective analysis of all pregnant women undergoing POCUS for suspected appendicitis between June 2010–June 2020 in a tertiary emergency department. The primary outcome was to establish sensitivity, specificity, and likelihood ratios of POCUS in diagnosing acute appendicitis, overall and for each trimester. We used histology of the appendix as the reference standard in case of surgery. If appendectomy was not performed, the clinical course until childbirth was used to rule out appendicitis. If the patients underwent magnetic resonance imaging (MRI), we compared readings to POCUS.

**Results:**

A total of 61 women were included in the study, of whom 34 (55.7%) underwent appendectomy and in 30 (49.2%) an acute appendicitis was histopathologically confirmed. Sensitivity of POCUS was 66.7% (confidence interval [CI] 95% 47.1–82.7), specificity 96.8% (CI 95% 83.3–99.9), and positive likelihood ratio 20.7. Performance of POCUS was comparable in all trimesters, with highest sensitivity in the first trimester (72.7%). The MRI reading showed a sensitivity of 84.6% and a specificity of 100%. In the four negative appendectomies a MRI was not performed.

**Conclusion:**

Point-of-care ultrasound showed a high specificity and positive likelihood ratio in diagnosing acute appendicitis in pregnant women in all trimesters with suspected appendicitis. In negative (or inconclusive) cases further imaging as MRI could be helpful to avoid negative appendectomy.

## INTRODUCTION

Acute appendicitis is the most frequent non-obstetrical surgical emergency in pregnancy with a similar incidence as in the non-pregnant population (10/100,000).^1^ Misdiagnosis of an acute appendicitis in pregnancy may lead to adverse outcomes for both mother and child. Hence, a rapid diagnosis is of utmost importance.^2^ The incidence of complicated appendicitis is higher during pregnancy than in non-pregnant women and increases with gestational age. Severe complications may include early delivery, miscarriage, or stillbirth.^2^ Unlike in non-pregnant women, the clinical presentation during pregnancy is often non-specific, and there is a wide range of differential diagnosis, such as other gastrointestinal, genitourinary, or gynecological diseases.^1^,^3^ Ultrasound is most commonly used as the first imaging modality in the emergency setting. However, quality of sonographic imaging of the appendix is limited due to its shifting during pregnancy because of the growing gravid uterus, as well as by the clinician’s expertise.^1^ Literature is sparse regarding the diagnostic performance of point-of-care ultrasound (POCUS) for acute appendicitis in pregnant women.^4^,^5^ Our aim in this study was to determine performance criteria of POCUS for diagnosing acute appendicitis in pregnant women with clinically suspected appendicitis in the emergency department (ED).

## METHODS

This retrospective data analysis was conducted in a tertiary ED based on charts from patients who were admitted between June 2010–2020. The local ethics committee required written informed consent, as pregnancy is considered sensitive data (EKOS 20/116). We reported data according to the STARD 2015 (standards for reporting diagnostic accuracy studies) checklist.^6^

We screened the electronic health records of all pregnant women >16 years seen in our hospital within the study time for eligibility. Patients were included if they had received a POCUS examination for suspected appendicitis. Suspicion of appendicitis was clinically determined by the treating physician in the ED. All POCUS examinations were performed or supervised by attending emergency physicians (EP) trained in abdominal US, certified by the Swiss National Society of Ultrasound (SGUM).^7^ This certification includes three courses (basic, intermediate, and advanced) totaling 48 hours, as well as a final theoretical and practical exam. Prior to this, 500 abdominal US exams must be completed in a training program, 300 of which are under direct supervision. Abdominal scans include evaluation of the bowel and, therefore, the appendix.^8^ Exclusion criteria were lack of written informed consent or a missing written ultrasound report.

Our primary goal was to determine performance criteria of POCUS in diagnosing acute appendicitis in pregnant women (overall as well as for each trimester separately). The POCUS criteria for diagnosing acute appendicitis according to the SGUM are an appendix of >6 millimeters in diameter, absence of peristalsis, localized probe pressure pain, or an increased echogenicity of adjacent mesenteric fat.^9^,^10^ Absence of compressibility is also a criterion, as is the presence of an appendicolith, whereas hypervascularity in color Doppler is rarely applied.^9^ A case was counted as positive if one criterion was met, according to the clinician’s report. The attending visceral surgeon in charge was involved in each suspected case. All sonographic-determined appendicitis (positive) cases either underwent surgery, magnetic resonance imaging (MRI), or clinical follow-up according to the surgeon’s choice. If surgery was performed, the histopathological finding served as the control to determine appendicitis (reference standard in case of surgery). If the patient did not undergo surgery, an alternative diagnosis was given, and the uneventful clinical course until childbirth was used as the control to rule out appendicitis (reference standard in case of no surgery).

**Population Health Research Capsule**

What do we already know about this issue?

*Acute appendicitis is the most common non-obstetrical surgical emergency in pregnancy. Ultrasound is the imaging tool of choice, but its use is difficult during pregnancy*.

What was the research question?

*What is the diagnostic accuracy of point-of-care ultrasound (POCUS) in suspected appendicitis in pregnant women?*

What was the major finding of the study?

*Sensitivity of POCUS was 66.7% (95% CI 47.1–82.7), specificity 96.8% (CI 95% 83.3–99.9), and positive likelihood ratio 20.7, in all trimesters with suspected appendicitis*.

How does this improve population health?

*Quick bedside diagnosis of acute appendicitis in the ED leads to quick treatment, avoiding possibly serious fetal and maternal complications*.

We defined negative sonographic cases as either no signs of acute appendicitis (ie. normal appendix), or appendix not seen. Inconclusive cases (ie, appendix not visible) were not defined as a separate group due to the lack of a clear definition on a retrospective basis. Negative sonographic cases underwent MRI, surgery, or clinical follow-up (until childbirth) per the surgeon’s choice, appendicitis being confirmed or ruled out by histopathology or uneventful pregnancy as in positive sonographic cases. The same pathway was used for re-consultation, if any. If MRI was performed, we calculated diagnostic performance using the same reference standards as in US. The MRI was initially read or supervised by the attending radiologist on duty. Additionally, all MRI underwent a second look at the time of this study by a not-blinded senior radiologist (ie, knowing the first reading and the final diagnosis). Finally, we collected the data of all pregnant women undergoing appendectomy in the study period in our center to screen for women without US before surgery.

### Statistical Analysis

We calculated sensitivity, specificity, positive predictive value (PPV) and negative predictive value (NPV) as well as positive and negative likelihood ratio (LR) including 95% confidence intervals (CI) for ultrasound. Sensitivity and specificity were calculated for MRI as well. We presented continuous data as mean values ± SD or as median values with interquartile ranges, as appropriate. Categorical data were presented in percentages. We used SPSS version 25 software package (IBM Corporation, Armonk, NY) for statistical calculations.

## RESULTS

**Figure 1:**
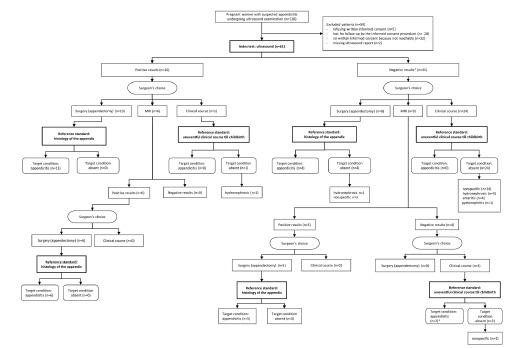
Patients’ inclusion flow chart and diagnostic STARD pathway. *Due to clinical deterioration both patients had an additional imaging study (one ultrasound, one magnetic resonance imaging) one day later, both with positive results. These two patients underwent surgery with histologically confirmed acute appendicitis. *STARD*, Standards for Reporting Diagnostic Accuracy; *MRI*, magnetic resonance imaging.

A total of 120 patients underwent US examination for suspected appendicitis, of whom 61 (50.8%) were included in the study. The inclusion chart and the diagnostic pathway for all patients are illustrated in Figure 1. Prevalence of histologically confirmed appendicitis was 49% (n = 30). The median age at the time of US was 31 years (range 21–40), gestational age was 17 weeks of gestation (WOG), and 13 WOG for histologically diagnosed appendicitis, respectively. Median pain duration was one day (range 1–2; Table 1).

On POCUS examination 20 cases were positive for acute appendicitis (ie, at least one of the above-described criteria was met), and 41 cases were negative. Of the 41 negative cases, the normal appendix could be visualized in eight cases and could not be visualized in 32 cases. None of the visualized normal appendixes resulted in a false negative examination. Sensitivity and specificity of US in diagnosing appendicitis was 66.7% (CI 95% 47.1–82.7) and 96.8% (CI 95% 83.3–99.9%), respectively. Sensitivity and specificity were comparable in all three trimesters, with best results in the second trimester (Table 2). There was no re-consultation; however, two patients were admitted for follow-up. One patient had a repeat ultrasound, and the other an MRI on the next day after admission. Both exams were suggestive for appendicitis, so that both patients underwent appendectomy. Appendicitis was confirmed histologically.

The first MRI reading revealed 11 acute appendicitis of 15 MRIs, yielding a sensitivity of 84.6% and a specificity of 100%. The second, not blinded, retrospective reading diagnosed two additional cases of appendicitis of the 15 MRIs, meaning that two MRI readings were initially false negatives. Hence, second reading increased the sensitivity to 100%.

In the study period, every pregnant woman with suspected appendicitis had an US examination before appendectomy. Alternative diagnosis to appendicitis were “nonspecific abdominal pain” (n = 19), hydronephrosis (n = 7), enteritis (n = 4), and pyelonephritis (n = 1) (Figure 1). Additional informations about surgery, course of the pregnancy, and delivery (newborn data included) are presented as supplemental file.

## DISCUSSION

This study showed a moderate sensitivity and an excellent specificity and positive LR of POCUS in diagnosing acute appendicitis among pregnant women in all trimesters of pregnancy. The median gestational age at time of appendicitis was 13 WOG, although most of the women were in the second trimester at time of admission. Our data is comparable to the literature, as multiple studies showed that acute appendicitis affects mostly the second trimester.^11^–^15^

### Ultrasound

Only a few studies analyzed the performance of US in pregnant women with suspected appendicitis. Sensitivity ranged from 50–100% and specificity from 95–100%. All these studies included a smaller number of patients than this study did, and likewise only a few of the patients included in published studies had a confirmed acute appendicitis. Three of those studies also included women who were primarily in the first and second trimester (with only a few in the third trimester).^3^,^16^–^18^ Studies with slightly larger cohorts than the present study exist; however, they only analyzed women who underwent appendectomy.^4^,^5^ Therefore, information about prevalence of acute appendicitis is missing, which results in incorrect calculation of predictive values, and especially affects specificity. A recent systematic review and meta-analysis showed low diagnostic accuracy for acute appendicitis in pregnant women, but most of the included studies were retrospective, with small sample sizes, unclear inclusion criteria, and with patients who had undergone surgery.^19^

Ultrasound has multiple advantages as an imaging tool in pregnancy compared to other tools such as MRI or computed tomography (CT). Sonography is easily available in the EDs of most developed countries,and can be performed with low costs and lack of ionizing radiation. However, a clinician experienced in sonography has to be at the patient’s side, unlike MRI or CT that allow remote reading and diagnosing. Visualization of the appendix due to the pregnant uterus is a limiting factor, which especially limits the use of US in the third trimester. Non-visualization rate ranges from 7–97%.^3^,^16^,^20^ This may be explained as follows: the level of expertise may vary from clinician to clinician between and within the various studies. In addition, because some studies with low numbers of patients, most of whom were examined in their first or second trimester (where US is known to be more accurate than in the third trimester^3^), the overall sensitivity may have been overestimated. By contrast, in our study the accuracy of US was comparable in all trimesters. Interestingly, sensitivity and specificity were even better in the third as compared to the first trimester, which contradicts the findings of the aforementioned studies. The reason for this remains unclear. It is important to emphasize that our data, unlike that of other researchers, suggests that POCUS can be used to diagnose acute appendicitis in the last trimester if performed by well-trained emergency physicians.

### Magnetic Resonance Imaging

Magnetic resonance imaging is a valuable tool for diagnosing acute appendicitis in pregnant women due to its lack of ionizing radiation when compared to CT, and with better visualization of the appendix (approaching 100%) when compared to sonography.^21^ Limitations of MRI are higher cost, longer examination time, and less availability compared to CT or sonography.^22^ We calculated the accuracy of MRI examination with a high sensitivity of 84.6% and a specificity of 100%. These findings are comparable to those of other studies.^23^ In five (33.3%) cases, US was negative for acute appendicitis. In all these cases the appendix itself was not visible; neither were there any indirect signs of acute appendicitis. However, MRI examination of these patients showed signs of an inflamed appendix that were confirmed by histology following surgery. Therefore, we conclude that the use of MRI may be of additional value in pregnant women who have clinical signs of appendicitis but negative US findings.

Two MRI examinations diagnosed no acute appendicitis, although the patients suffered from typical symptoms. Retrospective re-analysis of these two MRI examinations revealed signs of inflammation of the appendix. Due to clinical deterioration both women had repeat imaging studies one day later. One patient underwent US, the other a MRI. Both additional imaging studies revealed an acute appendicitis. Both patients underwent surgery, and in both cases a perforated appendicitis was successfully removed. It remains unclear why the first reading of the MRI images missed the diagnoses and, therefore, postponed adequate treatment with subsequent prolonged suffering. Hence, careful evaluation of MRI and double reading by experienced radiologists are crucial as perforation of the appendix might potentially have been avoided in these patients if there had been initial correct readings.

## LIMITATIONS

There are several limitations to this study. It has a retrospective design instead of a prospective randomization. However, we included patients undergoing US in suspected appendicitis, and it was not limited to patients who underwent surgery. Patient selection was clinically determined by the treating emergency physician in charge. Nevertheless, at least for patients who underwent surgery, we included consecutive patients in the study period (no patient had an appendectomy without prior US). Although the diagnostic evaluation was driven by the attending surgeon in charge, every patient had a follow-up until childbirth, so that misdiagnosis of appendicitis would be negligible. The rather small sample size may have influenced the results as well as the high exclusion rate (due to lacking written consent), which could have generated possible selection bias. Although each emergency physician was certified and trained for abdominal US, individual differences in experience may have existed. Further prospective studies with a larger cohort are needed to confirm our results, because of the still relatively large confidence intervals.

## CONCLUSION

US showed a high specificity in diagnosing acute appendicitis in pregnant women presenting with suspected appendicitis. This suggests that patients with positive US findings could directly undergo surgery without any further imaging workup. In negative cases, MRI examination might be helpful to avoid negative appendectomy.

## Supplementary Information



## Figures and Tables

**Table 1:** Patients’ characteristics.

Subject	Median (range)^*^, N (%)
Age (years)	31 (24–40)^*^
Gestational age (weeks) at time of US	17 (4–39)^*^
Pain duration time (days) at time of US	1 (1–2)^*^
MRI examination	15 (24.5)
Prevalence of acute appendicitis overall	30 (49.1)
1st trimester	9 (30.0)
2nd trimester	15 (50.0)
3rd trimester	6 (20.0)
Gestational age (weeks) at time of appendicitis	13 (5–38)^*^

*US*, ultrasound; *MRI*, magnetic resonance imaging.

**Table 2:** Performance criteria of ultrasound.

Trimester % (CI 95%)	Sensitivity % (CI 95%)	Specificity	PPV % (CI 95%)	NPV % (CI 95%)	LR+ n (CI 95%)	LR− n (CI 95%)
All trimesters N = 61	66.7 (47.1–82.7)	96.8 (83.3–99.9)	95.2 (74.1–99.3)	75.0 (64.3–83.3)	20.7 (3–144.5)	0.3 (0.2–0.6)
1st trimester n = 16 (26%)	62.5 (24.5–91.5)	100 (63.1–100)	100	72.7 (52.2–86.7)	n/a	0.4 (0.2–0.9)
2nd trimester n = 34 (56%)	68.8 (41.3–89.0)	94.44 (72.7–99.9)	91.67 (61.4–98.7)	77.3 (62.0–87.6)	12.37 (1.8–85.5)	0.3 (0.2–0.7)
3rd trimester n = 11 (18%)	66.7 (22.3–95.7)	100 (47.8–100)	100	71.4 (44.6–88.6)	n/a	0.3 (0.1–1.0)

*CI*, confidence interval; *PPV*, positive predictive value; *NPV*, negative predictive value; *LR*, likelihood ratio; *n/a*, not available.
