# Supplementary material for: Accuracy of Point-of-care Ultrasound in Diagnosing Acute Appendicitis During Pregnancy
Source: West J Emerg Med. 2022 Oct 23;23(6):913–8. doi: 10.5811/westjem.2022.8.56638 (PMC9683773; doi:10.5811/westjem.2022.8.56638)
Supplement: Supplementary file 1 [file wjem-23-913-s001.docx]

**Supporting information**

We analyzed as secondary outcomes the rates of surgical access by laparoscopy and laparotomy, perioperative complications (using the Clavien-Dindo classification of surgical complications)^1^, and the histopathological findings of the appendix (descriptive analysis). Rates of fetal loss, premature uterine contractions, application of tocolysis, induction of fetal lung maturation, preterm delivery, gestational age at time of delivery, mode of delivery, APGAR score, arterial umbilical pH value of the newborn, and its birth weight were determined in both groups (appendicitis vs no-appendicitis).

Differences between proportions were calculated with the two-tailed student t-test, Mann-Whitney-U-test, or the Fisher’s exact test, as appropriate. P-values < 0.05 were considered statistically significant.

**RESULTS**

**Surgery and histopathological findings of the appendix**

Appendectomy was performed in 34 (55.7%) of the pregnant women. Of these, 27 (79.4%) underwent laparoscopic and 7 (20.5%) underwent open appendectomy, respectively. One (2.9%) of the 7 open approaches was initially started as a laparoscopic and then converted to an open procedure because of adhesions. Thirty (88.2%) appendices showed signs of acute appendicitis in histopathological findings: 10 (33.3%) cases showed a perforation, and 19 (63.3%) cases exhibited signs of a local inflammatory reaction (periappendicitis or local peritonitis). A beginning inflammation was seen in 1 (3.3%) case. In 4 (11.7%) cases histopathological findings showed no signs of acute appendicitis: 1 (2.9%) finding was an appendix with an endometriosis, the other 3 (8.8%) appendices appeared normal upon microscopic analysis. In these cases, US was negative, a MRI was not performed, and the indication for appendectomy was a clinically suspected appendicitis. In 1 case the appendectomy was performed during the cesarean section (39 WOG), the other 3 appendectomies were at 13, 20 and 28 WOG. None of the 34 resected appendices showed signs of malignancy. Two (5.8%) patients had grade II complications according to the Clavien-Dindo classification. One of these two had a paralytic ileus, while the other one had a laparotomy wound infection that was treated successfully with intravenous antibiotics. Both patients had a perforated appendicitis treated by open approach.

**Fetal loss**

No fetal loss occurred in the appendicitis group. In the no appendicitis group (NA) a total of 4 fetal losses were registered (1 spontaneous abortion, 2 induced abortions and 1 stillbirth). However, all losses happened independently from the suspected appendicitis and occurred later in pregnancy.

**Premature uterine contractions, tocolysis, fetal lung maturation, and preterm delivery**

As specific internal guidelines for this rare event were not implemented, the decision for or against tocolysis was made individually for each case. In 4 out of 5 cases within the appendicitis group (n=30) the tocolysis was given prophylactically during appendectomy. Hexoprenaline was given in 1 case followed by 48 hours of atosiban due to immediate tachycardia. Only 1 woman received nifedipine for 3 days (20mg orally three times a day). Indomethacine was given in 1 case and in 1 case the type of tocolysis was not documented.

Only 1 (3.3%) woman started having contractions directly after appendectomy, and tocolysis was therefore started (atosiban) postoperatively. The other 2 (6.7%) women in the appendicitis group had tocolysis weeks later (without any connection to the appendicitis) due to premature contractions (33 and 25 WOG; 1 atosiban, 1 nifedipine and atosiban).

The only tocolysis in the NA group (n=31) was given because of premature contractions 9 weeks after the suspected appendicitis (nifedipine).

Of the 6 (20%) women in the appendicitis group who received corticosteroids for fetal lung maturation (betamethasone 2x12 mg IV), 1 patient had received the first dose abroad, before repatriation to Switzerland. The treatment was completed in our hospital (second application of steroids after 24 hours). Only 1 patient started having contractions perioperatively, and therefore the first dose was given. Corticosteroids were given in 1 case prophylactically one day after surgery, because the clinical condition initially worsened. Later the woman recovered fully and gave birth at 39+1 weeks of gestation (WOG). In 1 case it was started preoperatively due to doctor’s choice. A number of 2 women in the appendicitis group received corticosteroids later in pregnancy (at 30 and 25 WOG) due to premature contractions. In the NA group in 1 case fetal lung maturation was given weeks later at 33 WOG due to premature contractions, and in the other case at 33 WOG due to cervical shortening.

In the appendicitis group, 1 (3.3%) woman started having contractions directly after appendectomy and went into preterm labor despite tocolysis (33 WOG, atosiban).

In summary, there were no significant differences in the two groups (appendicitis vs no-appendicitis) related to the pregnancy clinical course, child and birth data (Table S1, S2).

**Table S1**: secondary outcomes. No. = number. SD = standard deviation.

| **Patients, No.**  **(N=61)** | **Appendicitis** | **No Appendicitis** | **p-value** |
| --- | --- | --- | --- |
|  | **(N=30, 49%), n (%)** | **(N=31, 51%), n (%)** |  |
| Fetal loss |  |  |  |
| Abortion, spontaneous | 0 | 1 (0.3) | 1 |
| Abortion, induced | 0 | 2 (0.7) | 0.49 |
| Stillbirth | 0 | 1 (0.3) | 1 |
| Premature uterine contractions | 3 (10.0) | 2 (6.5) | 0.67 |
| Tocolysis |  |  |  |
| Any indication during pregnancy | 7 (23.3) | 1 (3.2) | 0.053 |
| of which perioperative | 5 (71.4) | - | - |
| Induction of fetal lung maturation |  |  |  |
| Any indication during pregnancy | 6 (20.0) | 1 (3.2) | 0.053 |
| of which perioperative | 4 (66.7) | - | - |
| Preterm delivery | 2 (6.7) | 3 (9.7) | 1 |
| Gestational age; weeks, mean (SD) | 39+2 (+/- 45.8 days) | 39+0 (+/- 41.9 days) | 0.88 |
| Twin pregnancies | 2 (6.6) | 2 (6.4) | 1 |
| Mode of delivery |  |  |  |
| Spontaneous delivery | 16 (50.0) | 12 (41.4) | 0.61 |
| Vacuum extraction | 6 (18.8) | 4 (13.8) | 0.73 |
| Cesarean delivery | 10 (31.3) | 13 (44.8) | 0.3 |

**Table S2**: child data. No. = number. IQR = interquartile range.

| **Children, No.**  **(N=61)** | **Appendicitis** | **No Appendicitis** | **p-value** |
| --- | --- | --- | --- |
|  | **(N=32, 52%),**  **median (IQR)** | **(N=29, 48%),**  **median (IQR)** |  |
| APGAR 1 min | 9 (7.5-9) | 8 (7-9) | 0.45 |
| APGAR 5 min | 9 (8.5-10) | 9 (8-10) | 0.57 |
| APGAR 10 min | 10 (9-10) | 10 (9-10) | 0.63 |
| Arterial umbilical cord (pH) | 7.3 (7.17-7.31) | 7.28 (7.19-7.32) | 0.91 |
| Birth weight (g) | 3510 (3034-3823) | 3170 (2918-3465) | 0.13 |

**DISCUSSION**

**Surgery and histopathological findings of the appendix**

79% of the appendectomies were performed by a LA. Previously it has been argued that laparoscopy is contradicted in pregnancy due to pneumoperitoneum (and its possible reduced perfusion of the foetus) and the risk of uterine/foetal injury due to trocar insertion. However, new evidence shows that LA is safe in all trimesters and clinical outcomes are equivalent to an OA, while having all advantages of LA (less postoperative pain, less postoperative ileus, decreased length of hospital stays, and faster return to work).^2^ Our study supports (although not statistically significant) this evidence: both postoperative complications and the preterm delivery directly after appendectomy appeared after OA, no complications appeared in the LA group. Initial abdominal access in the LA can be safely done with Verres needle or open access if the fundal height is taken into account.^2^

Negative appendectomy rate was calculated with 12%, which is lower than described in literature (36%).^3^ The higher negative appendectomy rate in pregnant women compared to non-pregnant patients seems linked to reduced preoperative imaging. ^3^ In our study, 44% of the patients had an ultrasound and a MRI examination, this may explain the lower appendectomy rate in our study. All 4 patients with negative appendectomy (negative histology) had an ultrasound examination with non-visualization of the appendix. Surgery was performed upon doctor’s choice due to the clinical presentation without further imaging (as MRI). If MRI were performed and a normal appendix were seen, surgery might have been avoided and reduced the negative appendectomy rate even more. No fetal or maternal complications were seen in women, who underwent negative appendectomy.

A relatively high amount of perforated appendicitis was seen in our study (33%), this supports findings in literature, whereas perforated appendicitis occurs more often in pregnancy due to delayed diagnosis.^4^

**Foetal loss**

Although acute appendicitis is widely known to be associated with increased foetal mortality, in our study foetal loss only occurred in the NA group; the spontaneous abortion was 6 weeks after the ultrasound, which makes a correlation very unlikely. The induced abortions and the stillbirth occurred independently to the initial suspicion of appendicitis.

**Tocolysis, induction of fetal lung maturation and preterm delivery**

A tocolysis was started dependent on the consultant as a lack of standard operative procedure in our institution. In 4 cases it was started prophylactically, in 1 case due to premature contractions perioperatively. Only little literature exists about perioperative tocolysis. A systematic review published in 2008 found no statistically significant difference in the rate of preterm delivery postoperatively between women with or without prophylactic tocolysis.^5^ To our knowledge the SAGE guideline (Society of American Gastrointestinal and Endoscopic Surgeons) is currently the only guideline available suggesting that tocolysis should not be used prophylactically; it should be considered, when signs of preterm labor are present perioperatively. In general, tocolysis should be limited to 48 hours.^2^

Corticosteroids for fetal lung maturation were also given as of doctor’s choice. Only in 1 case it was given completely prophylactically without premature contractions (only the first dose, as surgery could not be delayed for 48 hours), the other 3 women received it, either because it had already been started abroad or because of the presenting clinic. There is no consensus in literature: in most cases, surgery cannot be delayed for 48 hours for the two or four doses of corticosteroids required depending on the regimen used. Hence, induction of fetal lung maturation can be evaluated in rare cases, if the clinic allows a delay for 48 hours.^6^

Only one of the aforementioned appendicitis-related complications in pregnancy was seen in our study: 1 woman went into labour directly after appendectomy at 33 WOG. She presented with a perforated appendicitis, which is known to be associated with an increased risk of preterm delivery (and should therefore be treated with imminent priority). The histological finding was a massive ulcerous-phlegmonous necrotic appendicitis.

**Birth and child data**

This study showed no statistically significant results related to childbirth: gestational age at time of delivery was comparable (39+2 vs. 39+0 WOG), which supports findings of a huge register study, whereas the risk of preterm delivery is increased the week after appendectomy with no further increase, if the pregnancy continues. No increase was seen in the number of stillbirths after appendectomy, which also supports the findings of the register study. However, in the register study the birth weight was lower in the appendectomy group (78 +/- 24g) which could not be supported by our findings.^7^

**REFERENCES**

1. Dindo D, Demartines N, Clavien PA. Classification of surgical complications: a new proposal with evaluation in a cohort of 6336 patients and results of a survey. *Ann Surg*. Aug 2004;240(2):205-13.

2. Yumi H, Surgeons GCotSoAGaE. Guidelines for diagnosis, treatment, and use of laparoscopy for surgical problems during pregnancy: this statement was reviewed and approved by the Board of Governors of the Society of American Gastrointestinal and Endoscopic Surgeons (SAGES), September 2007. It was prepared by the SAGES Guidelines Committee. *Surg Endosc*. Apr 2008;22(4):849-61.

3. Ito K, Ito H, Whang EE, et al. Appendectomy in pregnancy: evaluation of the risks of a negative appendectomy. *Am J Surg*. Feb 2012;203(2):145-50.

4. Aggenbach L, Zeeman GG, Cantineau AE, et al. Impact of appendicitis during pregnancy: no delay in accurate diagnosis and treatment. *Int J Surg*. Mar 2015;15:84-9.

5. Walsh CA, Tang T, Walsh SR. Laparoscopic versus open appendicectomy in pregnancy: a systematic review. *Int J Surg*. Aug 2008;6(4):339-44.

6. Juhasz-Böss I, Solomayer E, Strik M, et al. Abdominaleingriffe in der Schwangerschaft – eine interdisziplinäre Herausforderung. *Dtsch Arztebl International*. July 7, 2014 2014;111(27-28):465-72.

7. Mazze RI, Källén B. Appendectomy during pregnancy: a Swedish registry study of 778 cases*. Obstet Gynecol*. Jun 1991;77(6):835-40.
